# Prevalence of Intestinal Parasites among Rural Inhabitants of Fouman, Guilan Province, Northern Iran with Emphasis on *Strongyloides stercoralis*

**Published:** 2020

**Authors:** Meysam SHARIFDINI, Laleh GHANBARZADEH, Ameneh BARIKANI, Mehrzad SARAEI

**Affiliations:** 1. Department of Parasitology and Mycology, School of Medicine, Guilan University of Medical Sciences, Rasht, Iran; 2. Cellular and Molecular Research Center, Qazvin University of Medical Sciences, Qazvin, Iran; 3. Department of Medical Parasitology and Mycology, School of Medicine, Qazvin University of Medical Sciences, Qazvin, Iran; 4. Children Growth Research Center, Qazvin University of Medical Sciences, Qazvin, Iran

**Keywords:** Intestinal parasites, Prevalence, *Strongyloides stercoralis*, *Trichostrongylus*, Iran

## Abstract

**Background:**

Intestinal parasitic infections (IPIs) are among the most important etiologies of gastrointestinal disorders in developing countries. The present study was performed to determine the prevalence of IPIs in rural inhabitants of Fouman, northern Iran.

**Methods:**

Overall, 31 villages were randomly selected during 2015–2016. Stool samples were collected from 1500 inhabitants aged 2–87. The samples were examined by direct wet smear, formalin ethyl-acetate concentration and agar plate culture. Trichrome staining and modified acid-fast staining were used as confirmatory tests for intestinal amoeba and flagellates and *cryptosporidium* spp., respectively. Data were analyzed with Chi-Square and Fisher exact tests using SPSS.

**Results:**

8.06% of participants were positive for at least one intestinal parasite. The prevalence of mixed parasitic infections was 0.87%. The most prevalent IPIs were caused by *Trichostrongylus* spp. (3.13%), followed by *Strongyloides stercoralis* (1.5%), *Giardia lamblia* (1.3%), and *Entamoeba coli* (1.0%), *Blastocystis hominis* (0.86%), *E. histolytica/dispar* (0.53%), *Endolimax nana* (0.26%), *Iodamoeba butschlii* (0.13%), *Trichuris trichiura* (0.07%), *Enterobius vermicularis* (0.07%), Hook worm (0.07%) and *E. hartmani* (0.07%). Statistically, the prevalence of IPIs showed significant differences regarding the age groups, education status, occupation (*P*<0.001), and the habit of eating raw vegetables (*P*<0.007), whereas, the differences were insignificant with regard to sex (*P*=0.924) and water supply (*P*=0.088).

**Conclusion:**

The prevalence of IPIs, especially soil-transmitted helminthes (STHs) has sharply decreased in northern Iran. Excluding *Trichostrongylus* spp*.* and *S. stercoralis*, other intestinal parasites only produce a marginal and unnoticeable health problem in this area, today.

## Introduction

Intestinal parasitic infections (IPIs) are the most prevalent infections among the world’s population with significant morbidity and mortality rate (1). More than a quarter of people are infected with these parasites throughout the world and IPIs result in 450 million illnesses, especially in developing countries and 58 million children, suffer from these infections every year (2–4). In poor and developing countries, IPIs are known as a major public health problem. On the other hand, in developed countries, some intestinal parasites are still important causes of gastrointestinal disorders (5).

These infections are reported to cause several complications including growth retardation in children, iron deficiency anemia, and other physical and mental health problems (6, 7). The infections by intestinal parasites are associated with low socioeconomic levels, poor environmental sanitation, and lack of access to clean drinking water and food (8, 9).

These infections are most common in rural population due to high exposure to parasitic agents (10). Soil-transmitted helminthes (STHs), including *Ascaris lumbricoides*, *Trichuris trichiura*, hookworms and *Strongyloides stercoralis* are among the most important groups of intestinal infectious agents causing major health problems, of which *S. stercoralis* is the most neglected parasite (11, 12). Nowadays, the prevalence of human geohelminthes, have sharply decreased in Iran (13) while in some parts of the country, the incidence of *S. stercoralis* is high as a result of several factors including humid climate, local customs and ability of autoinfection in its life cycle (11, 12, 14, 15).

Several studies have been conducted on the prevalence of IPIs in rural areas of northern Iran, but there is limited updated epidemiological information about these infections especially in Guilan Province that is an endemic area for *S. stercoralis* (12, 14–16).

The aim of this study was to determine the prevalence of IPIs with emphasis on *S. stercoralis* among the rural residents of Fouman district, located in Guilan Province, northern Iran using a population-based study that provided a clear picture from the current status of infections in this area.

## Materials and Methods

### Ethical approval

This study protocol was approved by the Ethics Committee of Qazvin University of Medical Sciences, (Ref. No. IR.QUMS.REC.1394.111).

### Study area

**Fig. 1:**
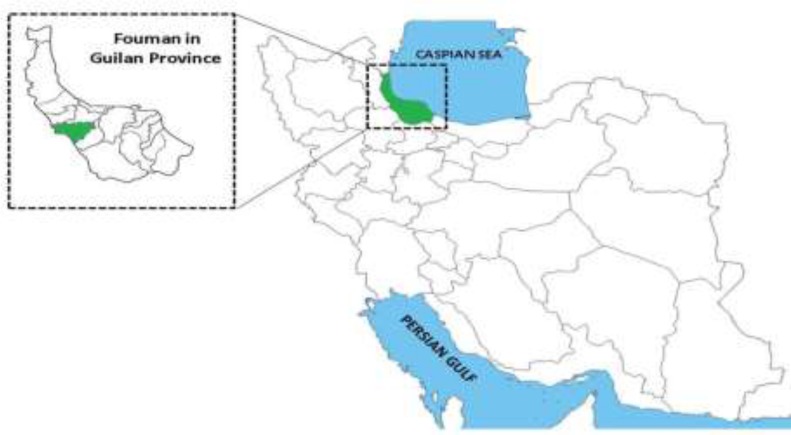
The study area. Fouman is located in southwestern part of Guilan province in northern Iran and the southern part of Caspian Sea (16)

This study was conducted in villages of Fouman located in southwestern part of Guilan Province in northern Iran and in southern part of Caspian Sea. The area of Fouman is about 1002 square km and has 156 villages with a rural population of 62561 people. It is situated between the orbits of 48° and 52′ to 49° and 27′ of east longitude from Greenwich meridian and circuits 37° and 1′ to 37° and 17′ of north longitude from the equator (Fig. 1). This area has a humid subtropical climate. This region of the province is geographically divided into two parts, including, plain zone and mountainous forest zone. The most common jobs of rural inhabitants are rice and tobacco farming in the plain zone and animal husbandry in the mountainous zone (17).

### Sampling and stool collection

This cross-sectional epidemiological study was carried out on rural inhabitants of Fouman during 2015–2016. Of 161 villages, 31 were randomly selected, based on the geographical distribution map. The demographic data of the study area was obtained from the Health Division of Guilan University of Medical Sciences. The sample size was estimated at 1500 specimens (considering *P*=1.0%, α=0.05, d=5.0%, and level of confidence=95.0%). Multistage sampling procedure was used for collecting specimens.

### Stool examinations

Diarrheic stool samples were examined by direct wet-mount smears and others tested by formalin ethyl-acetate concentration method. Moreover, the samples suspected of having *Cryptosporidium* spp. were tested with modified acid-fast staining technique. The Gomori’s trichrome staining technique was used as a confirmatory test for suspicious samples infected with *Blastocystis* spp., intestinal amoebae, and flagellates.

### Agar plate culture

In addition, all samples were subjected to agar plate culture technique for the detection of *S. stercoralis* described previously (18, 19). In brief, 3–4 gr of fecal sample was put on nutrient agar plate incubated at 28–30 °C for 2–3 days. Plates were evaluated by stereomicro-scope for detecting any larva and adults or their tracks. For collecting larvae, the surface of the positive agar plate was washed out by lukewarm phosphate-buffered saline (PBS) solution (18, 19). Differentiation of *S. stercoralis* from other probable nematode larvae including *Trichostrongylus* spp., hookworm, and free-living nematodes was performed based on morphological characteristics (20).

### Data analysis

Statistical analysis was performed by Chi-square and Fisher exact tests using SPSS software version 16 while a *P-*value of <0.05 was considered significant, statistically.

## Results

Overall, 1500 rural inhabitants were included, 653 (43.5%) male, and 847 (56.5%) female. Among the total participants, 121 (8.06%) were positive for at least one intestinal parasite by four parasitological techniques that identified the individuals with helminthic 66 (4.4%) and protozoan 52 (3.46%) parasites. Mixed infections were observed in 13 individuals (0.87%). The most prevalent IPIs were *Trichostrongylus* spp. (3.13%), *S. stercoralis* (1.5%), *Giardia lamblia* (1.3%), and *Entamoeba coli* (1.0%) (Table 1).

**Table 1:** Prevalence of infectivity with different species of intestinal parasites in 1500 inhabitants of rural areas of Guilan province, northern Iran (2015–2016)

***Intestinal parasites***		***N***	***%***
Single	*Giardia lamblia*	18	1.2
	*Entamoeba coli*	12	0.8
	*Entamoeba histolytica/dispar*	6	0.4
	*Endolimax nana*	1	0.07
	*Blastocystis hominis*	8	0.53
	*Trichostrongylus* spp.	42	2.8
	*Strongyloides stercoralis*	20	1.33
	*Enterobius vermicularis*	1	0.07
Double	*Blastocystis hominis + Entamoeba coli*	1	0.07
	*Blastocystis hominis+ Entamoeba hartmani*	1	0.07
	*Blastocystis hominis + Endolimax nana*	1	0.07
	*Entamoeba coli + Iodamoeba butschlii*	1	0.07
	*Entamoeba coli+ Entamoeba histolytica/dispar*	1	0.07
	*Entamoeba histolytica/dispar + Trichostrongylus* spp.	1	0.07
	*Trichostrongylus* spp. *+ Strongyloides stercoralis*	3	0.2
Triple	*Giardia lamblia + Blastocystis hominis+ Endolimax nana*	1	0.07
	*Blastocystis hominis + Endolimax nana + Iodamoeba butschlii*	1	0.07
	*Giardia lamblia +* Hook worm*+ Enterobius vermicularis*	1	0.07
	*Entamoeba coli + Trichostrongylus* spp. *+ Trichuris trichiura*	1	0.07

The distribution of infectivity with intestinal parasites in the population according to sex, age group, educational status, occupation, raw vegetable consumption, and water supply status is illustrated in Table 2. Some of these demographic factors including age (*P*<0.001), educational status (*P*=0.001), occupation (*P*<0.001), and raw vegetable consumption (*P*=0.006) were significantly associated to IPIs. Other demographic characteristics such as sex and water supply did not show any significant difference regarding the prevalence of infection with IPs (Table 2). Out of the 1500 individuals examined, 23 (1.5%) and 17 (1.1%) cases were infected with *S. stercoralis* using agar plate culture and formalin ethyl-acetate concentration, respectively. Agar plate culture technique detected all cases identified as positive by formalin ethyl-acetate concentration.

**Table 2:** Prevalence of infection with intestinal parasites in rural areas of Guilan province, northern Iran according to the demographic factors (2015–2016)

***Variables***	***Positive***	***Negative***	***OR (CI95%)***	**P-*value***
	***N (%)***	***N (%)***		
Age group (yr)				*P*<0.001
<10	7 (2.9)	233 (97.1)	Reference	-
10–20	10 (4.5)	213 (95.5)	1.56(0.58–4.17)	=0.51
20–30	16 (8.7)	168 (91.3)	3.17(1.27–7.87)	<0.01
30–40	19 (6.6)	267 (93.4)	2.36(0.97–5.73)	<0.07
40–50	23 (8.6)	243 (91.4)	3.15(1.32–7.48)	<0.009
50–60	21 (12.7)	144 (87.3)	4.85(2.01–11.71)	<0.001
>60	25 (18.4)	111 (81.6)	7.49(3.14–17.86)	<0.001
Gender				*P*= 0.924
Male	52 (8)	601 (92)	Reference	-
Female	69 (8.1)	778 (91.9)	1.02(0.7–1.49)	
Educational status				*P*<0.001
Illiterate	45 (14.1)	275 (85.9)	Reference	-
Pre school	3 (3.6)	81 (96.4)	0.22(0.06–0.74)	0.007
Primary school	53 (6.6)	750 (93.4)	0.43(0.28–0.65)	<0.001
Secondary school	17 (7.2)	220 (92.8)	0.47(0.26–0.84)	<0.01
Collage and above	3 (5.4)	53 (94.6)	0.34(0.1–1.15)	0.09
Occupation				*P*<0.001
Shepherd	8 (38.1)	13 (61.9)	Reference	-
Farmer	17 (14.4)	101 (85.6)	0.02(0.001–0.49)	0.005
Gov’t employer	0 (0)	31 (100)	0.27(0.09–0.75)	0.03
Worker	17 (12.1)	124 (87.9)	0.22(0.08–0.61)	<0.01
Student	10 (3.2)	298 (96.8)	0.05(0.01–0.16)	<0.001
House wife	54 (9.1)	538 (90.9)	0.16(0.06–0.41)	<0.001
Other	15 (5.2)	274 (94.8)	0.08(0.3–0.24)	<0.001
Raw vegetable consumption				*P*=0.006
Daily	28 (14.5)	165 (85.5)	Reference	-
Once a week	79 (7.5)	970 (92.5)	0.47(0.3–0.76)	0.01
Once a month	11 (6.4)	160 (93.6)	0.4(0.19–0.84)	0.01
Rarely	3 (4.2)	69 (95.8)	0.25(0.07–0.87)	0.02
Never	0 (0)	15 (100)	0.19(0.01–3.38)	0.6
Water supply status				*P*= 0.088
Untreated (well, mineral spring)	32 (6.3)	472 (93.7)	Reference	-
Treated pipe water	89 (8.9)	907 (91.1)	1.44(0.95–2.20)	

Therefore, this method showed the ability to detect the parasite 1.35 times higher than the formalin ethyl-acetate concentration procedure. The prevalence of infection with *S. stercoralis* according to sex, age group, educational status, and occupation is shown in Table 3. A statistically significant difference was found between the age and occupation of the individuals infected with *S. stercoralis* (*P*<0.001). The distribution of infection with *Trichostrongylus* spp. in the study population according to sex, age group, educational status, and occupation is illustrated in Table 4. There was a significant difference between the prevalence of infection and the age group (*P*=0.001), occupation (*P*<0.001), and educational status (*P*=0.01) of the people infected with *Trichostrongylus* spp.

**Table 3:** Prevalence of infection with *S. stercoralis* in rural areas of Guilan province, northern Iran according to the demographic factors (2015–2016)

***Variables***	***Positive***	***Negative***	***OR (CI95%)***	**P-*value***
	***N(%)***	***N(%)***		
Age group (yr)				*P*<0.001
<30	0 (0)	674(100)	Reference	
≥ 30	23 (2.7)	830 (97.3)	37.35(2.26–616.4)	
Gender				*P*=0.526
Male	12 (1.8)	641 (98.2)	Reference	
Female	11 (1.3)	836 (98.7)	1.42(0.62–3.24)	
Educational status				*P*=0.056
Illiterate	12 (3.8)	308 (96.2)	Reference	
Pre school	0 (0)	84 (100)	0.15(0.008–2.61)	P=0.45
Primary school	9 (1.1)	794 (98.9)	0.29(0.12–0.69)	P=0.01
Secondary school	2 (0.8)	235 (99.2)	0.21(0.048–0.98)	P=0.49
Collage and above	0 (0)	56 (100)	0.22(0.01–3.93)	0.83
Occupation				*P*<0. 001
Shepherd	4 (19)	17 (81)	Reference	
Gov’t employer	0 (0)	31 (100)	0.06(0.003–1.37)	0.22
Farmer	4 (3.4)	114 (96.6)	0.14(0.03–0.65)	0.02
Worker	2 (1.4)	139 (98.6)	0.06(0.01–0.35)	0.005
Student	0 (0)	308 (100)	0.006(0.0003–0.13)	<0.001
House wife	11 (1.9)	581 (98.1)	0.08(0.023–0.27)	0.002
Other	2 (0.7)	287 (99.3)	0.02(0.005–0.17)	<0.001

**Table 4:** Prevalence of infection with *Trichostrongylus spp.* in rural areas of Guilan province, northern Iran according to the demographic factors (2015–2016)

***Variables***	***Positive***	***Negative***	***OR (CI 95%)***	**P-*value***
	***N (%)***	***N (%)***		
Age group (yr)				*P*=0.001
<10	1 (0.4)	239 (99.6)	Reference	
10–20	4 (1.8)	219 (98.2)	4.36(0.48–39.35)	0.32
20–30	7 (3.8)	177 (96.2)	9.45(1.15–77.51)	0.02
30–40	6 (2.1)	280 (97.9)	5.12(0.61–42.83)	0.19
40–50	11 (4.1)	255 (95.9)	10.31(1.32–80.45)	0.009
50–60	7 (4.2)	158 (95.8)	10.59(1.29–86.88)	<0.01
>60	11 (8.1)	125 (91.9)	21.03(2.68–164.8)	<0.001
Gender				*P*=0.98
Male	21 (3.2)	632 (96.8)	Reference	
Female	26 (3.1)	821 (96.9)	0.95(0.53–1.71)	
Educational status				*P*=0.01
Illiterate	20 (6.3)	300 (93.8)	Reference	
Pre school	0 (0)	84 (100)	0.08(0.005–1.49)	0.09
Primary school	20 (2.5)	783 (97.5)	0.38(0.20–0.72)	0.005
Secondary school	6 (2.5)	231 (97.5)	0.38(0.15–0.98)	0.058
Collage and above	1 (1.8)	55 (98.2)	0.27(0.03–2.07)	0.3
Occupation				*P*<0.001
Shepherd	5 (23.8)	16 (76.2)	Reference	
Gov’t employer	0 (0)	31 (100)	0.05(0.002–1.0)	0.09
Farmer	7 (5.9)	111 (94.1)	0.2 (0.05–0.71)	0.03
Worker	8 (5.7)	133 (94.3)	0.19(0.056–0.65)	0.02
Student	5 (1.6)	303 (98.4)	0.05(0.013–0.20)	<0.001
House wife	21 (3.5)	571 (96.5)	0.11(0.039–0.35)	0.002
Other	1 (0.3)	288 (99.7)	0.01(0.012–0.1)	<0.001

## Discussion

The present study demonstrated that the prevalence of IPIs has sharply declined among the rural population of Guilan Province compared to the previous study performed in this region (21). In distant past, these parasites, especially STHs, were extremely prevalent in this province, so that at least 50% of rural inhabitants were infected with the parasites (21). In recent decades, the prevalence of IPIs has significantly dropped in Guilan Province (13). The following factors have synergistically affected the situations, leading to a remarkable reduction in the number of these infections:

(I) Establishment of rural health houses and improvement of primary health care (PHC): Some of the important tasks performed by the health houses are the education of public health, promotion of community participation, and diseases control services (22). (II) Expansion of rural roads and increase in the number of medical graduates led to promotion of PHC services in these areas. (III) Sanitary disposal of human excreta: In the distant past, the rural toilets in northern Iran were mostly unsanitary (21). (IV) Increased literacy, education level, and health knowledge: Illiterates are usually unaware of transmission routes of IPs and also, less inclined to comply with health recommendations. The literacy rate has remarkably increased in Iran during three recent decades (23). (V) Media such as TV and radio have played a positive role in the prevention of IPIs in Iran. Over four decades ago, TV was not available for rural residents in many villages of the country and radio was the only commonly used and available social medium for these people. Currently, TV is almost accessible to all villagers throughout the country (24). (VI) Easy access to anti-parasitic drugs: Prior to the establishment of health houses, anti-parasitic drugs were not easily available for the rural residents of Iran. At present, anthelmintic agents are easily available to these people through health houses, even in the most remote areas of the country.

In the current study, the prevalence of helminthic infections (4.4%) was slightly higher than the protozoan infections (3.46%), while the results of most studies conducted in Iran in recent years are in contradiction with our findings (25–30). These differences may be related to demographic and geographic differences. 37.5% of inhabitants of rural areas of Boyer-Ahmad district, Southwestern Iran were infected with IPs among those *G. lamblia* with a prevalence rate of 17.46% was the most pathogenic protozoan. In addition, no one was infected with intestinal helminthes (25). About 56% of inhabitants and tribes of Chelgerd, located in Chahar-Mahal and Bakhtiari Province in southwest Iran, were positive for IPs with prevalence rates of 55.1% and 0.9% for intestinal protozoan and helminthic parasites, respectively (29). 29.3% of rural individuals in Jiroft, located in Kerman Province southeast Iran, were infected with IPs and the prevalence of protozoan and helminthic infections were 27.4% and 1.8%, respectively (26). 32.2% of patients with gastrointestinal disorders in Nahavand County, a region located in western Iran, were infected with IPs and the prevalence rates for intestinal helminthes and protozoa were 0.38%, and 32%, respectively (27). In Rasht, the capital of Guilan Province, *S. stercoralis* was the only helminthic infection with a prevalence rate of 1.2%, while the protozoan infections showed a prevalence rate equal to 28.3% (14). Among the schoolchildren aged 7–14 in Sari, northern Iran, the prevalence rates of protozoan, helminthic, and mixed infections were 23.2%, 5.1%, and 5.0%, respectively (28).

Our results showed a sharp decline in the prevalence of human STHs, as there was no case of infection with *A. lumbricoides* and only one case of *T. trichiura* and one case of hookworm were detected. By contrast, *Trichostrongylus* spp., which was the most prevalent IPIs in our study, is mostly transmitted by infected animals, because livestock is the main reservoir of the parasite in the Middle East, in particular in Iran (30–33). The zoonotic is the main route for transmission of the parasite in northern Iran. In fact, the traditional breeding of cattle and sheep is very common in this area and these animals graze almost freely in pastures contaminated with the feces of livestock. In addition, there are local wild and self-growing plants such as *Eryngium campestre*, locally called Chochagh (Sheshakh), that grow in these areas. The plants may be consumed as raw which may be a risk factor for trichostrongyliasis (34). On the other hand, the feces of livestock may be used as fertilizer for agricultural and gardening purposes, which can be a potential zoonotic source for transmission of the parasite.

In this study, infection with IPs did not show a significant difference between males and females, which is in agreement with the findings of previous studies (15, 26, 29, 30). Based on our findings, the prevalence of IPIs in older people (age ≥30) was higher than in young people (age <30), which is similar to the findings reported previously (35). It was possibly due to low education level of older people and living in conditions with poor sanitation. On the other hand, high exposure to IPs sources as a result of adult outdoor activities may justify higher prevalence of IPIs among this population. Moreover, the higher prevalence of *S. stercoralis* infections in older individuals could probably be another reason for this finding (36).

Guilan and Mazandran provinces in northern Iran and Khuzestan Province in the south are the most important foci of *S. stercoralis* as well as hookworms in Iran (18, 19, 21, 37). In our study, *S. stercoralis* was the second common parasite in the study area, and the prevalence of this parasite was remarkably higher than hookworms, whereas the picture was quite different in relatively distant past (21). This difference may be related to the following three major factors: (I) Autoinfection and long-term sustainability of *S. stercoralis* infections: this parasite is the only STHs that has controlled internal autoinfection in immuno-competent subjects in whom it causes chronic infection, which remains for a long period of time (38, 39). Thus, the initial infection in a number of subjects may date back years ago. In our study, 69.5% of *S. stercoralis* infections were observed in people over 50 yr and it was not detected in those below 30 yr (II) Zoonotic transmission: *S. stercoralis* has been reported in some animals such as dogs (40), which may have played as a reservoir for transmission to human. (III) Having a free-living cycle: *S. stercoralis* is the only STHs that may develop into free-living adult worms under optimal climatic conditions. This ability can help the survival and environmental reproduction of the parasite and therefore increases potential transmissibility to human.

This study has illustrated that *S. stercoralis* infection was more prevalent in individuals over 30 yr of age. It was in agreement with most other studies found that prevalence of strongyloidiasis is more prevalent in people of old age (19, 37). This issue is related to its ability to autoinfection in infected individuals for several decades or even whole life (19). Similar to previous studies (18, 41, 42), the results of present study confirmed that the agar plate culture method is more sensitive than formalin ethyl-acetate concentration in the diagnosis of *S. stercoralis*.

## Conclusion

The prevalence of IPIs, especially STHs, including *A. lumbricoides*, *T. trichiura*, and human hookworms has sharply decreased in northern Iran. The health problems related to these parasites have significantly reduced in the study area not considered as major health problems anymore. However, *Trichostrongylus* spp. as a zoonotic infection and *S. stercoralis* as an opportunist infection are still regarded as important health challenges associated with IPIs in rural inhabitants of Guilan Province.
